# Fibrinogen-to-albumin ratio links systemic inflammation to osteoporotic fractures and shows discriminative performance together with PLR, NLR, and MLR

**DOI:** 10.5937/jomb0-64875

**Published:** 2026-06-16

**Authors:** Youxin Fu, Daigui Cao, Zeyu Liu, Zhiwei Liu, Jianping Guo, Xu Zhou, Jie Hao

**Affiliations:** 1 Chongqing Medical University First Affiliated Hospital, Department of Orthopedics, Chongqing, China; 2 Chongqing General Hospital, Department of Orthopedics, Chongqing University, Chongqing, China

**Keywords:** fibrinogen albumin ratio, inflammatory markers, osteoporotic fractures, predictive model, odnos fibrinogena i albumina, inflamatorni markeri, osteoporotični prelomi, prediktivni model

## Abstract

**Background:**

Osteoporotic fracture (OPF) remains a major cause of disability in older adults. Laboratory-available composite indices integrating coagulation, inflammation, and nutritional status may facilitate risk stratification in patients with osteoporosis. To investigate the relationship between the fibrinogen-to-albumin ratio (FAR) and systemic inflammatory indices (platelet-to-lymphocyte ratio [PLR], neutrophil-to-lymphocyte ratio [NLR], and monocyte-to-lymphocyte ratio [MLR]) in osteoporosis, and to evaluate their discriminative value for identifying concurrent osteoporotic fractures.

**Methods:**

This retrospective study included 98 patients with osteoporosis admitted between January 2024 and May 2025, categorized into a fracture group (n=39) and a non-fracture group (n = 59). FAR, PLR, NLR, and MLR were calculated from routine laboratory tests obtained within 24 h of admission. Group differences were assessed, Pearson correlation was used to examine associations between FAR and inflammatory indices, and logistic regression was performed to identify independent clinical/imaging correlates of fracture. Receiver operating characteristic (ROC) curves were constructed to evaluate discriminative performance.

## Abstract (sr)

**Uvod:**

Osteoporotični prelom (OPF) ostaje glavni uzrok invaliditeta kod starijih osoba. Laboratorijski dostupni kompozitni indeksi koji integrišu koagulaciju, upalu i nutritivni status mogu olakšati stratifikaciju rizika kod pacijenata sa osteoporozom. Cilj je ispitati vezu između odnosa fibrinogena i albumina (FAR) i sistemskih inflamatornih indeksa (odnos trombocita i limfocita [PLR], odnos neutrofila i limfocita [NLR] i odnos monocita i limfocita [MLR]) kod osteoporoze i proceniti njihovu diskriminativnu vrednost za identifikaciju istovremenih osteoporotičnih preloma.

**Metode:**

Ova retrospektivna studija obuhvatila je 98 pacijenata sa osteoporozom primljenih između januara 2024. i maja 2025. godine, kategorisanih u grupu sa prelomima (n=39) i grupu bez preloma (n=59). FAR, PLR, NLR i MLR su izra~unati iz rutinskih laboratorijskih testova dobijenih u roku od 24 sata od prijema. Procenjene su razlike između grupa, korišćena je Pirsonova korelacija za ispitivanje povezanosti između FAR i inflamatornih indeksa, a izvršena je logistička regresija za identifikaciju nezavisnih kliničkih/slikovnih korelata preloma. Konstruisane su ROC krive (Receiver Operating Characteristic) za procenu diskriminativne performanse.

**Rezultati:**

FAR, PLR, NLR i MLR bili su značajno viši kod pacijenata sa osteoporozom komplikovanom prelomom nego kod onih bez preloma (sve P&lt;0,001). FAR je pokazao pozitivne korelacije sa PLR, NLR i MLR (sve P&lt;0,001). U multivarijantnoj analizi, starost &gt;60 godina, lumbalno-dorzalna fascijalna povreda i znak vakuumske vertebra bili su nezavisno povezani sa pojavom preloma (sve P&lt;0,05). ROC analiza je pokazala visoke diskriminativne performanse za FAR, PLR, NLR i MLR, sa AUC od 0,900.

**Zaključak:**

FAR i inflamatorni odnosi leukocita/trombocita bili su povišeni kod osteoporotičnih pacijenata sa prelomima i bili su pozitivno međusobno povezani, što podržava laboratorijski fenotip upale-ishrane-koagulacije kod OPF. Ovi rutinski laboratorijski indeksi pokazali su snažnu diskriminativnu efikasnost u identifikaciji istovremenih osteoporotičnih preloma i mogu pomoći u laboratorijskoj stratifikaciji rizika u kliničkoj praksi.

## Introduction

Osteoporotic fracture (OPF) is among the most prevalent and serious skeletal disorders in clinical practice, with a high global incidence, particularly in older adults. OPF not only causes pain and functional impairment that substantially diminish quality of life, but also increases susceptibility to complications, thereby elevating disability and mortality and imposing a considerable burden on families and society [1]. Accordingly, identifying robust risk predictors and developing accurate prediction models are critical for the early prevention and intervention of OPF [2].

The fibrinogen-to-albumin ratio (FAR) has recently gained attention as a composite marker reflecting both systemic inflammation and nutritional status. Fibrinogen, an acute-phase reactant, rises markedly during inflammatory responses, whereas albumin, a key indicator of nutritional status, typically decreases in the setting of inflammation or physiological stress. By integrating these two parameters, FAR may more comprehensively capture the interplay between inflammatory activity and nutritional reserve. Prior studies have shown that FAR is closely associated with the occurrence and prognosis of various diseases [3].

Similarly, the platelet-to-lymphocyte ratio (PLR) and neutrophil-to-lymphocyte ratio (NLR) are widely used inflammatory indices that reflect the presence and magnitude of systemic inflammatory responses and have been linked to disease development, treatment response, and prognosis across a range of conditions, including malignancies, infectious diseases, and autoimmune disorders. The monocyte-to-lymphocyte ratio (MLR) has also been implicated in the initiation and progression of inflammation. In the pathogenesis of OPF, inflammatory mediators may disrupt bone remodeling, promoting bone loss and increasing fracture susceptibility [4]. Therefore, these inflammation-related indices may be associated with OPF.

However, evidence regarding the relationships between FAR and PLR, NLR, and MLR and OPF remains limited and lacks systematic evaluation. Moreover, effective OPF risk prediction models incorporating these indices have not yet been established [5]. The present study aimed to further elucidate the associations of FAR and inflammatory markers (PLR, NLR, and MLR) with OPF and to develop a risk prediction model, thereby providing a theoretical basis for early warning and personalized prevention of OPF and offering more valuable references for clinical practice.

## Materials and methods

### Study design and patients

A retrospective analysis was performed on 98 patients with osteoporosis admitted to our hospital between January 2024 and May 2025. The inclusion criteria were as follows: (1) all patients met the diagnostic criteria for primary osteoporosis [6]; (2) relevant laboratory parameters, including fibrinogen, albumin, platelet count, lymphocyte count, neutrophil count, and monocyte count, were measured within 24 h after admission; (3) patients in the fracture group experienced their first osteoporotic fracture; and (4) complete clinical data were available. The exclusion criteria were: (1) severe dysfunction of vital organs such as the heart, liver, or kidneys; (2) hematologic diseases; (3) infectious diseases or autoimmune inflammatory diseases within the previous month; and (4) pregnancy or lactation. Patients were divided into a fracture group (n=39) and a non-fracture group (n=59) according to the presence of fracture.

The associations of the fibrinogen-to-albumin ratio (FAR) and inflammatory markers with osteoporotic fractures were compared between the two groups. Univariate and multivariate logistic regression analyses were conducted to identify factors associated with fracture occurrence among patients with osteoporosis. Receiver operating characteristic (ROC) curve analysis was used to evaluate the predictive value of FAR and inflammatory markers for osteoporotic fractures. The variables included age (≥60 years, <60 years), sex (male, female), smoking history (yes, no), alcohol consumption (yes, no), comorbid hypertension (yes, no), comorbid diabetes mellitus (yes, no), comorbid hyperlipidemia (yes, no), lumbar-dorsal fascial injury (yes, no), vertebral intravertebral vacuum cleft sign (present, absent), systolic blood pressure, diastolic blood pressure, platelet count, white blood cell count, NLR, D-dimer, C-reactive protein, triglycerides, total cholesterol, high-density lipoprotein, low-density lipoprotein, and homocysteine.

Diagnostic criteria for lumbar-dorsal fascial injury were as follows: (1) symptoms—patients typically presented with low back pain, which could manifest as soreness or stabbing pain and was exacerbated by activity, particularly lumbar flexion/extension and rotation, with partial relief after rest; (2) physical signs—marked localized tenderness was present at the injured site, sometimes accompanied by palpable cord-like induration; increased muscle tone was observed, and severe cases could exhibit restricted range of motion; and (3) medical history— often including excessive lumbar strain, acute sprain, or long-term poor posture. Diagnostic criteria for the vertebral vacuum cleft sign were: (1) imaging findings—on X-ray, CT, or MRI, a radiolucent cleft was observed within the vertebral body, typically presenting as a low-density area beneath the end- plate or within the vertebral body; and (2) clinical correlation—patients might present with low back pain and functional impairment, and some reported a history of minor trauma.

### Statistical analysis

Statistical analyses were performed using Statistic Package for Social Science (SPSS) version 26.0 (IBM, Armonk, NY, USA). Categorical variables are presented as n or percentages (%) and were compared using the c^2^ test. Continuous variables are expressed as mean ± standard deviation (SD) and were compared using the t test. Multivariate analysis was conducted using logistic regression. A two-sided P value < 0.05 was considered statistically significant. Receiver operating characteristic (ROC) curve analysis was used to establish the prediction model, and the area under the curve (AUC) was calculated to evaluate the predictive value of the fibrinogen-to-albumin ratio (FAR) and inflammatory markers (PLR, NLR, and MLR) for osteoporotic fractures.

## Results

### Association of the fibrinogen-to-albumin ratio and inflammatory markers with osteoporotic fracture

Patients with osteoporosis complicated by fracture had significantly higher levels of FAR, PLR, NLR, and MLR than those without fracture (t = 10.668, 28.926, 18.112, and 13.627, respectively; all P < 0.001). The results are shown in Table 1. Pearson correlation analysis demonstrated that FAR was positively correlated with PLR, NLR, and MLR (r = 0.428, 0.635, 0.716, and 0.529, respectively; all P < 0.001).

**Table 1:** Association of the fibrinogen-to-albumin ratio and inflammatory markers with Osteoporotic fracture.

Group	n	FAR	PLR	NLR	MLR
Fracture group	39	0.32±0.08	153.99±11.43	3.38±0.59	10.44±1.26
Non-fracture group	59	0.18±0.05	90.15± 10.19	1.84±0.23	7.08±1.15
*t*		10.668	28.926	18.112	13.627
*P*		<0.001	<0.001	<0.001	<0.001

### Univariate analysis of factors associated with fracture in patients with osteoporosis

Compared with the non-fracture group, the fracture group differed significantly in age, lumbar-dorsal fascial injury, and the vertebral vacuum cleft sign (all P < 0.05). No significant between-group differences were found for sex, smoking history, alcohol consumption, comorbid hypertension, diabetes mellitus, or hyperlipidemia; systolic or diastolic blood pressure; platelet or white blood cell count; NLR; D-dimer; C-reactive protein; triglycerides; total cholesterol; high-density lipoprotein; low-density lipoprotein; or homocysteine (all P > 0.05). These results are summarized in Table 2.

**Table 2:** Univariate analysis of factors associated with fracture in patients with osteoporosis.

Variables	n	Fracture group <br>(n=39)	Non-fracture group <br>(n = 59)	c^2^	*P*
Age, years				21.651	<0.001
≥60	40	27 (69.23)	13 (22.03)		
<60	58	12 (30.77)	46 (77.97)		
Gender				0.393	0.531
Male	44	16 (41.03)	28 (47.46)		
Female	54	23 (58.97)	31 (52.54)		
Smoking history				0.014	0.906
Yes	37	15 (38.46)	22 (37.29)		
No	61	24 (61.54)	37 (62.71)		
Alcohol consumption				0.082	0.775
Yes	41	17 (43.59)	24 (40.68)		
No	57	22 (56.41)	35 (59.32)		
Hypertension				0.809	0.368
Yes	53	11 (28.21)	12 (20.34)		
No	75	28 (71.79)	47 (79.66)		
Diabetes mellitus				0.406	0.524
Yes	34	15 (38.46)	19 (32.20)		
No	64	24 (61.54)	40 (67.80)		
Hyperlipidemia				0.014	0.906
Yes	27	11 (28.21)	16 (27.12)		
No	71	28 (71.79)	43 (72.88)		
Lumbar-dorsal fascial injury				9.231	0.002
Yes	42	24 (61.54)	18 (30.51)		
No	56	15 (38.46)	41 (69.49)		
Vertebral vacuum cleft sign				21.651	<0.001
Yes	40	27 (69.23)	13 (22.03)		
No	58	12 (30.77)	46 (77.97)		
Systolic blood pressure (mmHg)	98	155.40±3.46	154.51±3.51	1.236	0.219
Diastolic blood pressure (mmHg)	98	90.43±5.67	89.43±5.35	0.884	0.379
Platelet count (10^9^/L)	98	194.48±5.52	192.53±5.59	1.669	0.093
White blood cell count (10^9^/L)	98	11.02±1.46	10.46±1.35	1.946	0.055
NLR	98	11.15±1.40	10.75±1.32	1.433	0.155
D-dimer	98	0.82±0.16	0.79±0.18	0.843	0.401
C-reactive protein (mg/L)	98	15.61±1.69	15.48±1.70	0.371	0.711
Triglycerides (mmol/L)	98	1.49±0.22	1.42±0.26	1.385	0.169
Total cholesterol (mmol/L)	98	4.45±0.42	4.31±0.51	1.424	0.158
HDL-C (mmol/L)	98	0.78±0.14	0.81±0.16	0.954	0.342
LDL-C (mmol/L)	98	4.50±0.57	4.28±0.52	1.973	0.051
Homocysteine (mmol/L)	98	23.40±2.44	23.75±2.57	0.673	0.503

### Multivariate logistic regression analysis of factors associated with fracture in patients with osteoporosis

The occurrence of fracture in patients with osteoporosis was defined as the dependent variable. Variables that were statistically significant in the univariate analysis were entered as independent variables and were quantitatively coded (Table 3). Multivariate logistic regression analysis showed that age ≥60 years, lumbar-dorsal fascial injury, and the vertebral vacuum cleft sign were independent risk factors for fracture in patients with osteoporosis (all P < 0.05) (Table 4).

**Table 3:** Coding of independent variables.

Variable	Coding
Age	≥60=1, 60=0
Lumbar-dorsal fascial injury	Yes=1, No=0
Vertebral vacuum cleft sign	Yes=1, No=0

**Table 4:** Multivariate logistic regression analysis of factors associated with fracture in patients with osteoporosis.

Risk factor	*β*	*SE*	Wald c^2^	*P* Value	*OR*	95%*CI*
Age ≥60 years	1.486	0.312	22.684	0.000	4.419	2.39-8.146
Lumbar-dorsal fascial injury	1.528	0.286	28.544	0.000	4.609	2.631-8.073
Vertebral vacuum cleft sign	1.506	0.305	24.381	0.000	4.509	2.480-8.197

### Predictive value of the fibrinogen-to-albumin ratio and inflammatory markers in patients with osteoporotic fracture

**Figure 1:**
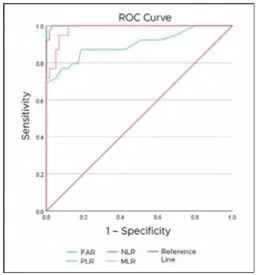
ROC curves of the fibrinogen-to-albumin ratio and inflammatory markers for predicting osteoporotic fractures.

Receiver operating characteristic (ROC) curves were constructed using the predicted probability as the test variable and fracture status (presence vs. absence) as the state variable. The areas under the ROC curve (AUCs) for FAR, PLR, NLR, and MLR were 0.900, 1.000, 0.998, and 0.983, respectively (Figure 1).

## Discussion

Osteoporotic fracture is a condition that seriously threatens the health and quality of life of middle-aged and older adults, and its incidence has been increasing annually. Accurate early prediction of fracture risk enables timely and effective interventions to slow disease progression [7]. In recent years, increasing attention has been paid to blood-based biomarkers for estimating osteoporotic fracture risk [8]. The fibrinogen-to-albumin ratio (FAR) and inflammatory indices— platelet-to-lymphocyte ratio (PLR), neutrophil-to-lymphocyte ratio (NLR), and monocyte-to-lymphocyte ratio (MLR)—have demonstrated value in inflammatory responses and prognostic assessment across various diseases; however, evidence regarding their association with osteoporotic fractures and their clinical utility remains limited [9].

Fibrinogen is an acute-phase reactant primarily involved in inflammation and coagulation. Albumin, a liver-synthesized plasma protein, reflects nutritional status and systemic inflammatory burden. FAR, defined as the fibrinogen-to-albumin ratio, integrates information on both inflammation and nutrition. Patients with osteoporotic fractures often exhibit a chronic inflammatory state, with increased fibrinogen levels; concurrently, albumin may decrease due to inflammation-related consumption and other factors, resulting in an elevated FAR. A high FAR may promote bone loss and increase fracture risk by disrupting the bone metabolic microenvironment, enhancing osteoclast activity, and inhibiting osteoblast function [10].

As inflammatory biomarkers, PLR, NLR, and MLR represent the relative proportions of platelets, neutrophils, and monocytes to lymphocytes, respectively. Lymphocytes are important in immune regulation and bone metabolism, whereas platelets, neutrophils, and monocytes play active roles in inflammatory responses. In patients with osteoporotic fractures, inflammatory activation may shift these cellular proportions [11]. For example, an increased NLR reflects neutrophilia and lymphopenia and is associated with inflammatory cytokine release; these cytokines can impair osteocyte and osteoblast function and disturb bone metabolic homeostasis. PLR and MLR may act through similar pathways, and their elevation may likewise contribute to the development and progression of osteoporotic fractures.

In the present study, FAR, PLR, NLR, and MLR were higher in patients with osteoporosis complicated by fracture than in those without fracture. Pearson correlation analysis further showed that FAR was positively correlated with PLR, NLR, and MLR. ROC curve analysis yielded AUCs of 0.900 for FAR, 1.000 for PLR, 0.998 for NLR, and 0.983 for MLR, indicating good predictive performance for osteoporotic fracture. This may be explained by inflammation-related increases in platelet, neutrophil, and monocyte counts and a relative decrease in lymphocytes, thereby elevating PLR, NLR, and MLR. Collectively, these changes reflect a higher inflammatory burden and enhanced cytokine release, which may disrupt bone metabolism, promote bone resorption, and inhibit bone formation [12].

Univariate and multivariate logistic regression analyses identified age ≥60 years, lumbar-dorsal fascial injury, and the vertebral vacuum cleft sign as independent risk factors for fracture in patients with osteoporosis. Possible explanations are as follows: (1) With advancing age, dysregulation of bone metabolism becomes more prominent. Osteoblast activity and bone-forming capacity decline, whereas relatively preserved osteoclast activity promotes bone resorption, leading to progressive bone loss and increased skeletal fragility [13]. In addition, decreased sex hormone levels and altered calcium-regulating hormones in older individuals impair calcium absorption and utilization, further exacerbating osteoporosis. Older adults also commonly have multiple chronic comorbidities and age-related functional decline; poorer balance and slower reflexes increase the risk of falls during daily activities, thereby elevating fracture risk [14]. (2) The lumbar-dorsal fascia provides support and protection for the spine and paraspinal musculature. Once injured, normal force transmission and spinal stability are compromised. To maintain posture and movement, increased activation of the lumbar musculature is required, which augments compressive loading on the vertebral bodies [15]. In patients with osteoporosis, vertebrae are structurally weakened and may be unable to tolerate these loads, predisposing them to compression deformity and fracture [16]. (3) The vertebral vacuum cleft sign reflects an intravertebral pathological change in osteoporotic vertebrae. A cleft disrupts vertebral integrity and biomechanics, markedly reducing load-bearing capacity [17]. As a result, the affected vertebra develops a structural weak point and is more likely to fracture during routine activities or with minor external forces. Moreover, the vertebral vacuum cleft sign often suggests more severe osteoporosis and poorer bone quality; even modest stress may exceed the vertebral tolerance threshold and precipitate fracture. These findings are consistent with reports by Ni Huifang [18], Qin Weicheng [19], and Liu Hui [20].

In summary, FAR and inflammatory markers (PLR, NLR, and MLR) are markedly elevated in patients with osteoporotic fractures and demonstrate good predictive value, providing clinically relevant evidence for the diagnosis and management of osteoporosis complicated by fracture. Nevertheless, the sample size of this study may be relatively small, and the study population may be subject to regional and demographic limitations, which could reduce the representativeness of the findings and limit their generalizability to all patients with osteoporotic fractures.

## Dodatak

### Conflict of interest statement

All the authors declare that they have no conflict of interest in this work.
